# Sleep Duration, Body Mass Index, and Dietary Behaviour among KSU Students

**DOI:** 10.3390/nu15030510

**Published:** 2023-01-18

**Authors:** Nora Alafif, Nawaf W. Alruwaili

**Affiliations:** Department of Community Health Sciences, College of Applied Medical Sciences, King Saud University, Riyadh 11433, Saudi Arabia

**Keywords:** sleep duration, BMI, dietary behaviour, fruits and vegetables

## Abstract

Background: Adolescents who receive an adequate amount of sleep benefit from a positive health status. Previous studies have documented several health consequences connected with obesity as well as short sleep duration among adolescents. Poor sleep quality with obesity and uncontrolled diet can lead to chronic diseases in the future. This study aimed to examine the link between eating habits, sleep duration, and body mass index (BMI) among King Saud University (KSU) students. Methods: The study was cross-sectional and conducted from February to May 2021 on 311 recruited students (male and female) of KSU premises. Pittsburgh Sleep Quality Index questionnaire was used to describe sleep duration linked with a dietary pattern that included fruit and vegetable intake. The questionnaire consists of two sections of 15 and 10 questions each. The questionnaire was created using the Google Forms tool and distributed through social media platforms like Twitter and WhatsApp. The obtained data was transferred into excel to perform the statistical analysis. Results: The mean total of students who participated in this study was 21.45 ± 23.11. Female students (72.3%) were actively involved in this study. About 30.2% of students were found to be overweight and obese. Around 67.8% of students had insufficient sleep, 32.2% had adequate sleep, and over 70% of students fell asleep within 30 min of going to bed. A total of 71.7% of students showed good sleep quality, whereas 28.3% reported poor sleep quality. BMI was categorized into four groups: 17.7% of individuals were underweight, 52.1% were of normal weight threshold, 20.6% were overweight, and 9.6% were obese. On a regular basis, 12.5% of students consume vegetables and 6.4% fruits daily. The results of this study show that only 8% of students eat breakfast, whereas 62.1% eat lunch, and 29.9% eat dinner. Conclusion: This study concludes that short sleep duration was associated with obesity among KSU students. This association was also found between sleep duration and dietary factors, specifically in the consumption of fruits and vegetables in terms of eating behaviour.

## 1. Introduction

Sleep is defined as a natural transitory condition of rest in which an individual is physically inactive and ignorant of their surroundings and many of their bodily functions [1]. The National Sleep Foundation recommends 7–9 h of sleep every night for individuals in their optimum working years (i.e., 18–25 years of age) to assist in the preserving and sustaining of age [2]. Adequate sleep duration, sleep timings, regularity, and the lack of sleep duration all play a role in attaining healthy sleep for optimal health. Type 2 diabetes, obesity, and cardiovascular disease are cardio metabolic health problems that can be exacerbated by insufficient sleep length and poor sleep quality, among adults between 18–60 years [3]. Poor sleep quality (SD) is a typical symptom of obesity. The most common form of obesity-related sleep problem, obstructive sleep apnea (OSA), is itself a risk factor for a number of diseases and disorders [4]. Despite this, not only do people with extreme obesity suffer from SD and OSA, but people with moderate obesity do as well, suggesting that factors other than the fat excess that causes recurrent narrowing and closure of the upper airway may also contribute to disturbed sleep. Increased visceral adipose tissue has been linked to the release of inflammatory cytokines, which may interfere with the body’s natural sleep-wake cycle. It has been suggested that one’s diet plays a part in controlling their sleeping patterns. The quality of sleep appears to suffer when carbohydrates are consumed in large quantities. Certain foods, including milk, fish, fruit, and vegetables, have been shown to have sleep-inducing qualities [5]. Several studies have shown a link between poor sleep and weight gain in the different age groups of participants, such as adults, adolescents, and children. A modification of the neuroendocrine control of hunger, typified by a drop in the levels of the anorexigenic hormone leptin and an increase in the levels of the orexigenic component ghrelin, is the most likely explanation to date of the sleep duration-body weight association. In this way, neuroendocrine alterations at work may ultimately encourage a surplus of calories and weight growth [6]. Adults need between seven and nine hours of sleep per night, according to sleep experts. Sadly, only about 65% of adults in the United States actually do this on a regular basis. In all eight TRE trials, participants’ sleep times were tracked using a combination of the Pittsburgh Sleep Quality Index (PSQI), a sleep diary, a subjective self-assessment survey, and accelerometers. No significant difference in sleep duration was observed in documented TRE trials. Most people were already getting at least 7 hours of sleep each night before the experiment began. Therefore, it should come as no surprise that healthy sleepers’ sleep duration did not change towards the end of the intervention. In the future, it will be interesting to see if TRE can increase sleep time for people who normally get less than 7 hours of sleep a night [7]. The World Health Organization (WHO) reports that 1.9 billion people were overweight, and 609 million were obese, in 2015 [8]. The body mass index (BMI) is used to classify overweight and obesity. BMI standards for children and adolescents differ depending on their age and gender. A BMI equal to or greater than 25 kg/m^2^ indicates an individual is overweight, and a BMI greater than 30 kg/m^2^ denotes obesity [9]. The first large-scale epidemiological study to analyze obesity/ BMI and sleep duration was published in 2000 and suggested that being fat was connected with less sleep. Since then, hundreds of studies in Paediatrica and adult populations have been published looking at the correlation between BMI and sleep time. However, BMI is widely used as an adiposity measure because it is simple to collect, analyze, and interpret across a variety of study contexts [10]. Obesity develops when the body stores excess fat as a response to insufficient physical activity or an unhealthy diet. Obesity as a human disease has finally been accepted after much debate. When the amount of energy taken in through food is more than the amount of energy burned off through exercise, the body stores the surplus as fat and the result is obesity. Obese people were divided into three age groups: (i) those between 0 and 4 years old, (ii) those between 5 and 19 years old, and (iii) those more than 19 years old. The cut-off for childhood obesity was determined to be a weight-to-height ratio of more than three standard deviations (SDs); the cut-off for overweight was two SDs. If a teen’s BMI was more than two SDs above WHO growth reference median, then they were considered obese; if their BMI was more than one SD above the WHO growth reference median. BMI is a typical numerical indicator of obesity. The International Obesity Task Force defines overweight as having a body mass index (BMI) over the 85th percentile and obesity as having a BMI above the 95th percentile. The prevalence of obesity has increased to alarming levels in Saudi Arabia, with 44% of females and 28% of males affected. Genetic polymorphisms are linked to obesity and overweight [8,9,10]. 

A change in traditional eating habits, an increase in a sedentary lifestyle, and a decrease in the prevalence of regular physical activity are some factors associated with the growing rates of overweight and obesity among Saudi children and adolescents [11]. Obesity has a wide range of physical health consequences, including cardiovascular disease, type 2 diabetes, sleep apnea, mental and developmental motor delays, and psychological problems [12].

The transition to university life is pivotal when young adults’ behaviours, especially their dietary habits, are amenable to change because of their increased autonomy in this domain. Inadequate nutritional intake and unhealthy eating behaviours are more likely to occur in these groups. The consumption of fried foods and a decrease in the consumption of fruits and vegetables are two examples of such practices. Poor workout regimens, lack of time management skills, and an increasing stress level due to schoolwork all play a role in weight gain [13]. Because dietary factors that affect sleep quality can be easily improved, assessing the relationship between sleep and food intake is relevant [14]. A comprehensive review and meta-analysis concluded that individuals who got less than six hours of sleep each night had a net positive energy balance of 385 kcal per day, which could lead to weight gain in the long run [15]. Prior studies have shown that sleep deprivation can lead to an increase in energy intake. This is because sleep deprivation leads to dysregulation of metabolic hormones leptin and ghrelin, critical regulators of hunger and satiety, and executive function involved in the internal regulation of food [16]. The imbalance between energy intake and expenditure is exacerbated by a lack of sleep, correlated to increased carbs, total sugar, total cholesterol, and saturated fat consumption. In addition, significant alterations in satiety-regulating hormones have been observed in individuals with short sleep duration, and these alterations occur independently of BMI [17,18]. Previous studies were carried out in many universities, including Saudi Arabia and outside its region. The current study was carried out during the COVID-19 pandemic. This study aims to identify the most common sleep hours and BMI classification among King Saud University (KSU) students and investigate the relationship between sleep duration, BMI, and dietary behaviour.

## 2. Materials and Methods

### 2.1. IRB Details

The Institutional Review Board at KSU approved this study (E-21-5822). All Saudi students who participated in this study signed the informed consent form.

### 2.2. Study Design

This study was designed as a cross-sectional study with 311 Saudi bachelor’s degree students at KSU in the Riyadh region.

### 2.3. Study Period

This study was conducted in 2021 to analyze the effects of sleep length on eating behaviours and BMI, and identify the most common BMI classification and sleep hours among KSU students from February 2021–May 2021. The study was carried out over three months. The inclusion criteria of study subject participants were Saudi students with a bachelor’s degree at KSU premises. Exclusion criteria included non-Saudi students, non-bachelor’s degree students, pregnant female students, students with a history of smoking or were currently smoking, and students with chronic diseases or psychological issues.

### 2.4. Detailed Protocol

All information was gathered through an online self-administered questionnaire that was generated in Arabic. The questionnaire was created using the Google Forms tool and distributed through social media platforms Twitter and WhatsApp. The details for the gain and loss of weight can be found in Supplemental File S1. 

### 2.5. Anthropometrics

Anthropometric measurements/data, including height, weight gain, and weight loss, were self-reported and obtained through the survey [8,10]. 

### 2.6. Questionnaire

In this questionnaire, we used some items from Pittsburgh Sleep Quality Index (PSQI) [19] relevant to sleep duration; for fruits and vegetables, we used some items from (eats all day), and Adolescent Friendly Health Clinics (AFHC) was used for sugar and fried food. Of the 36 close-ended questions developed based on previous studies [20], the first section included 15 questions about the student’s ethnic data. Additionally, the first section included questions about whether the participants were non-Saudi, had a non-bachelor’s degree, were suffering from any chronic diseases, were smoking, had any psychological problems or were on any psychological medications, and questions for females if they were pregnant and body weight change data. The second section included 10 questions about students’ eating behaviours. Finally, the third section consisted of 6 questions about students’ sleeping behaviour.

### 2.7. Statistical Analysis

Statistical analysis was carried out using SPSS. In addition, descriptive statistics were performed to accurately interpret and present the results of the characteristics of the participants and the responses of sleep duration, eating behaviours, and weight changes. Data were reported as frequencies and percentages. In addition, to investigate the association between sleep duration and weight gain, results at *p* < 0.05 were considered statistically significant.

## 3. Results

### 3.1. Sample Description

In this cross-sectional study, 311 students were recruited based on inclusion and exclusion criteria. The mean age of the total students involved in this study was 21.45 ± 23.11. The age criteria of the recruited students were reported to be 17–25 years, with 35.7% of students being between the ages of 17–20, 46% being between 21–22 years, and 18.3% being between 23–25 years of age. Female students were found to be 72.3% actively involved, while male students were found to be 27.7% involved. The recruited students were split into four groups: 30.2% were from the medical field, 20.6% were from the administrative field, 11.6% were from the science field, and 37.6% were from the humanity field. In this study, 25.4% of students belonged to the 1st–2nd and 5th–6th academic years, 42.1% belonged to 3rd–4th years students, and 7.1% belong to an internship. BMI categorization was divided into four groups of which 17.7% of students were classified in the underweight group, 52.1% in the normal weight criteria group, 20.6% fell in the overweight group, and 9.6% were assigned to the obese group. The demographic characteristics of the participants are shown in Table 1. 

### 3.2. Student’s Sleep Quality, Duration, and Sleeping Habits

Table 2 shows the sleep characteristics of the participants. In this study, 16.4% of students slept for less than 5 h per day, considered insufficient sleep, and 51.4% of students slept between 5–<7 h per day, which is classified as short sleep duration. Almost 20.9% of students slept for 7–9 h per day, whereas 11.3% slept more than 9 h per day. In this study, 23.1% of students fell asleep after 10 min, 27.1% within 20 min, 19.6% within 30 min, and 30.2% fell asleep more than 30 min later. The student’s sleep quality was categorized into four groups; 25.1% had very good sleep, 46.6% had a good sleep, 12.6% had poor sleep, and 15.7% had very poor sleep. The student’s sleep patterns in this study included 15.7% of them sleeping early and waking up early, 50.5% sleeping late at night and waking up early, whereas 30.9% of students slept late at night and woke up late in the morning, and 2.9% slept early and woke-up at late in the morning.

### 3.3. Sleeping Patterns and Dietary Habits

The current study results confirmed that 80.4% of the participants in this study were not adhering to any specific diet, while 10.3% were on an intermittent fasting diet, 3.9% were on a ketogenic diet, and 5.4% were on various types of diets, which included 3.5% as weight gain diet, 0.32% for a healthy diet and calorie deficit, and 0.64% for Mediterranean, as well as strict diets, as indicated in Table 3. However, there was no association between daily sleep hours and the large portion of their daily consumption of food items such as carbohydrates, vegetables, fruit, fast food, coffee, tea, meat, dairy products, soft drink, and energy drinks during the previous month.

**Figure 1:**
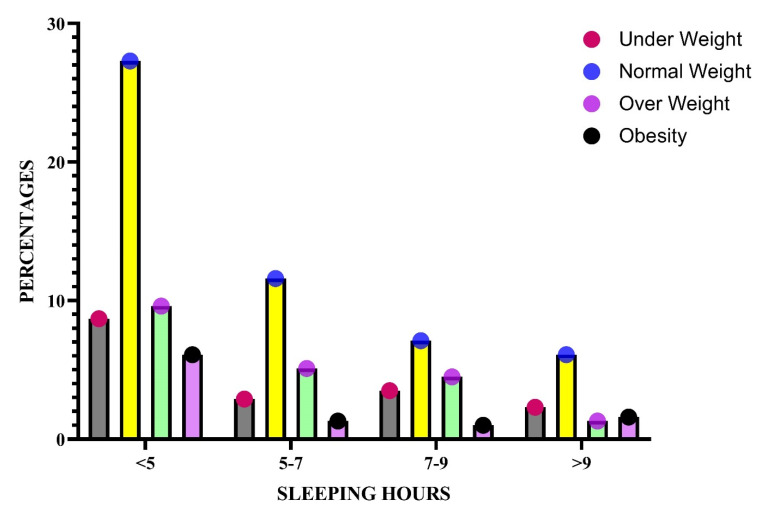
Sleeping patterns are characterized according to BMI group classification.

Moreover, 54.3% of the participants had no change in their appetite among different sleep duration groups. In addition, statistically, there was no difference between groups depending on their sleep hours (*p* = 0.11) and the time of consuming meals (*p* = 0.31). However, there was a correlation between sleep hours and weekly vegetable intake (*p* = 0.001, χ^2^ = 35.1), which indicates a statistically significant correlation between weekly intake of vegetable and sleep hours (Figure 1). In contrast, in those who ate fruit, there was no association between weekly intake of fruit and sleep duration, while there was a correlation between sleep quality and weekly fruit intake (*p* = 0.002, χ^2^ = 13.4).

## 4. Discussion

According to the descriptive study results, 20.6% of students were found to be overweight, while 9.6% were determined to be obese. A study by Aldahash et al. was consistent with our findings regarding the prevalence of overweight and obesity [21]. Overall, 30.2% of students were identified as overweight. One of the significant causes of weight gain is an imbalance between calorie consumption and physical activity. College students with obesity were more likely to have poor sleep duration and quality than their normal weight. Excess body weight can cause sleep problems due to disordered breathing, whereas sleep problems can cause weight gain due to abnormal metabolic and endocrine functioning. Both sleep duration and quality are essential for health, while sleep quality may be a better predictor. Previous studies have discovered an association between high BMI and short sleep duration, particularly duration and quality. The association between sleep and BMI in adolescents was complicated [22]. Previous global [23,24,25,26] and meta-analysis studies [27] indicate that shorter sleep duration is associated with higher BMI, with the strongest interaction occurring in women. However, certain studies were not related to this study [28]. However, studies by Sa et al. confirmed that obesity was associated with short and long sleep durations. The short sleep duration was recorded as <7 h/day, with >9 h/day considered a long sleep duration [23]. Some lifestyle factors, such as exercise, may improve sleep quality [14].

Two cross-sectional studies are consistent in their result regard positive correlation between both short and sleep duration and body mass index, the results of Krističević et al. [29] shows both short and long time spent in bed and poor sleep quality are associated with overweight/obesity status in young adults, also, in Lin et al. [30] studies, the result shows BMI was significantly higher in the participants who slept for a longer duration than in those who slept for a normal duration. No significant association was observed between sleep duration and overweight risk and both short and long sleep duration are associated with obesity risk. It should be noted that gender is an important factor should be considered, just like in this cross-sectional study [31], the prevalence of obesity in males was significantly higher than in Females, an obvious correlation was found between sleep quality and body mass index in females who took hypnotic drugs, and found that sleep quality in female students, but not in males, was associated with BMI. Moreover, the result of Meyer et al. [25] study shows that compared to young men, younger women were less likely to report sleeping less than 7 h/day and more likely to report sleeping more than 9 h/day, because that sleep duration was inversely associated with overweight, so short sleep duration was positively associated with BMI, overweight, and obesity in men, but not women. Similarly, the result of Sun et al. [32] shows short sleep duration (<6 h) was significantly associated with obesity among men, whereas longer duration was associated with obesity in women. One study was done in the U.S. and South Korea by Sa et al. [23] aimed to examine associations of sleep duration and quality with BMI-based weight status, The result shows that Koreans had higher overweight/obesity rates (59.4%) than blacks (51.5%) and whites (46.8%) when they use Asian BMI. However, Koreans had lower overweight/obesity rates (26.2%) when they use standard BMI, obesity was associated with both short (less than 7 h/night) and long sleep duration (more than 9 h/night) and poor sleep quality among all participants. Another cross-sectional study done in Saudi Arabia (Dahi Aldahash et al., 2018) shows a significant negative correlation was evident between BMI and sleep duration. Also, BMI was higher among students with short sleep duration. In the end, the result of the epidemiologic cohort Study shows short sleep is associated with higher body mass index, central adiposity, and increased markers of cardiovascular risk in adults and the elderly population [33]. short sleepers had a higher BMI when sleep duration was measured by Acti and PSG. There are no significant differences among sleep duration subsets when measured by the PSQIQ.

Fruits are consumed by 48.2% of students for at least 1–2 days a week, while vegetables are consumed by 31.8%. Compared to 17% of students who eat fruits, 28.9% consume vegetables 3–4 days per week. For 5–6 days each week, 18.3% of students consume fruits, and 4.2% consume vegetables. However, 24.1% and 8.4% of students who had never consumed fruits or vegetables were included in this study, respectively, while 12.5% of students consumed vegetables and 6.4% consumed fruits daily. In this study, 70.1% of students had a daily sugar intake, and almost half (45.3%) planned their breakfast daily. This survey shows that only 8% of students eat breakfast, 62.1% eat lunch, and 29.9% eat dinner. In this study, 67.8% of students had insufficient sleep, 32.2% had adequate sleep, and over 70% of students fell asleep within 30 min of going to bed. Students got a good sleep in 71.7% of cases, but 28.3% of students had problems sleeping at night. The current study revealed that while sugar and fried food had no association with sleep hours, there is a correlation between sleep hours and weekly vegetable intake, indicating a statistically significant correlation. These findings confirmed the previous studies showing that getting more sleep was related to eating more veggies and fruits [30,34,35]. In contrast, our study found no correlation between weekly fruit consumption and sleep duration. In our study, the majority of individuals (80.4%) did not follow any specific diet, and the majority of participants (54.3%) reported no change in their appetite across different sleep duration groups.

Poor eating habits and inadequate nutrients intake are very common among university students and poor sleep makes it worse, low consumption of fruits and high consumption of empty calories, and low-quality diet shown to be related to inappropriate sleep duration, thus sleep duration is an important factor in terms of good general health and quality of life and it’s related to some of health promotion behaviours. As evidence by many studies conducted in United States, Taiwan, and Korea. All these studies are cross-sectional except for one was a prospective cohort study. The aim of these studies to evaluate the effect of sleep duration on eating behaviour. Each of these studies has the same characteristics of the sample in terms of age group (>18 years), gender (male and female), also the same collected data in terms of self-reported anthropometrics, and sleep quality questionnaire, while the eating behavior questionnaire was different from study to another.There are two cross-sectional studies are consistent in their result regard positive correlation between sleep duration and eating behaviour in general, the results of Lin et al. [29] show the students with sleeping duration ≥ 7 h had a greater tendency to have good nutritional behaviour than those whose sleeping duration was <7 h, also in Quick et al. [36], the results show that who slept <8 h had significantly more negative eating attitudes (2% higher), poorer internal regulation of food (4% lower), and greater binge eating (4% higher) scores. It should be noted that gender is an important factor should be considered, just like in a cross-sectional study, the final results show among men subjects, the interaction between all three macronutrients consumed and sleep duration was not significantly associated with the risk of obesity but it was significantly associated in women, and for women who consumed high levels of protein or CHO and low level of fat, short sleep duration was associated with increased obesity risk [37]. In a cohort prospective study [38], the main finding was that the extent of snack dominance over meals in people with short sleep duration < 6 h as well as people with long sleep duration ≥ 10 h.

The strength of this study was that almost three-quarters of female students actively participated and one-quarter of male students were involved. With 311 participants, this cross-sectional study from a single university had an appropriate sample size after the pandemic. The main limitation of the present study is the lack of time, as the number of responses was limited due to the time constraints imposed on the study. Conversely, we received more female respondents than male respondents due to limited time. BMI is self-reported, which is another limitation of this study.

## 5. Conclusions

In Summary, short sleep duration was associated with obesity among KSU students. This association was also found between sleep duration and dietary factors, specifically in the consumption of vegetables in terms of eating behaviour. Future studies should be carried out in all Saudi universities around the nation, with increased attention on male students, to better understand sleep duration and the impacts of BMI and dietary behaviour. Long sleep duration studies are also recommended. The findings of this study could be used as supporting data for future studies to improve participants’ awareness of the relevance of sleep duration and its impact on BMI and eating behaviours. Anthropometric measurements should also be recorded at the clinic to avoid self-reported bias.

## Figures and Tables

**Table 1:** Demographic characteristics of the students’ participants.

	Categories	Number of Participants (%)
Age (years)	17–20 years 21–22 years 23–25 years	111 (35.7%) 143 (46%) 57 (18.3%)
Academic track	Medical track Administrative track Science track Humanity track	94 (30.2%) 64 (20.6%) 36 (11.6%) 117 (37.6%)
Academic year	1st–2nd year 3rd–4th year 5th–6th year Internship	79 (25.4%) 131 (42.1%) 79 (25.4%) 22 (7.1%)
Gender	Female Male	225 (72.3%) 86 (27.7%)
BMI (kg/m^2^)	Underweight Normal Overweight Obese	55 (17.7%) 162 (52.1%) 64 (20.6%) 30 (9.6%)

**Table 2:** Participants’ sleep characteristics.

	Categories	Number of Participants (%)
Hours of sleeping	<5 h 5–<7 h 7–9 h >9 h	51 (16.4%) 160 (51.4%) 65 (20.9%) 35 (11.3%)
Minutes to fall sleep	10 min 20 min 30 min >30 min	72 (23.1%) 84 (27.0%) 61 (19.6%) 94 (30.2%)
Sleep Quality	Very good Good Poor Very poor	78 (25.1%) 145 (46.6%) 39 (12.6%) 49 (15.7%)
Sleep pattern	Early wake-up–early sleep Early wake-up–late sleep Late wake-up–late sleep Late wake up–early sleep	49 (15.7%) 157 (50.5%) 96 (30.9%) 09 (2.9%)

**Table 3:** Students who followed different patterns of dietary behaviour.

Types of Diets	Total Number	Percentages
No diet	250	80.4%
Intermittent Fasting	32	10.3%
Keto Diet	12	3.9%
Weight Gain Diet	11	3.5%
Mediterranean Diet	02	0.64%
Healthy Diet	01	0.32%
Calorie Deficit	01	0.32%
Strict Diet	02	0.64%

## Data Availability

All the data is available in this manuscript.

## Supplementary Materials

The following supporting information can be downloaded at: https://www.mdpi.com/article/10.3390/nu15030510/s1, Supplementary File S1: Data for Body Weight.
