# Supplementary material for: Sleep Duration, Body Mass Index, and Dietary Behaviour among KSU Students
Source: Nutrients. 2023 Jan 18;15(3):510. doi: 10.3390/nu15030510 (PMC9918940; doi:10.3390/nu15030510)
Supplement: Supplementary file 1 [file nutrients-15-00510-s001.zip › nutrients-2130477-supplementary.pdf]

1. Body weight data:

1.1. Current weight (in kilograms): …………………

1.2. Height (in centimeters): ………………….

1.3. Did your weight change during quarantine?

- ○ Yes
- ○ No

1.4. If yes, did you gain or lose weight?

- ○ Gain
- ○ Lose

1.5. If you gained weight, how much did you gain approximately?

- ○ ≤ 1 kilogram
- ○ > From 1 – 3 kilograms
- ○ > From 3 – 5 kilograms
- ○ > 5 kilograms

1.6. If you lost weight, how much did you lose approximately?

- ○ ≤ 1 kilogram
- ○ > From 1 – 3 kilograms
- ○ > From 3 – 5 kilograms
- ○ > 5 kilograms
